# Editorial: New Trends in the Renin-Angiotensin-Aldosterone System in Cardiovascular Disease

**DOI:** 10.3389/fphar.2021.790439

**Published:** 2021-11-18

**Authors:** Ewa Szczepanska-Sadowska, Noreen F. Rossi

**Affiliations:** ^1^ Department of Experimental and Clinical Physiology, Medical University of Warsaw, Warsaw, Poland; ^2^ Departments of Internal Medicine and Physiology, Wayne State University School of Medicine, Detroit, MI, United States

**Keywords:** aldosteronism, angiotensins, COVID-19, heart failure, hypertension, ischemia

The special issue “New Trends in the Renin-Angiotensin-Aldosterone System in Cardiovascular Disease” presents studies of essential new achievements in the pathophysiology of the renin-angiotensin-aldosterone system (RAAS) relevant to treatment of cardiovascular diseases.

Starting from 1950s, a continuous flow of publications provided evidence for the complex organization of RAAS. Nonetheless, dysfunction of specific RAAS components in pathogenesis of cardiovascular diseases has not been satisfactorily explained. Currently, we understand RAAS as a powerful multifunctional entity which operates at the cellular level via specific renin, angiotensin and aldosterone receptors located in peripheral organs and the brain. Interplay of various components of RAAS with other systems is intensely analyzed. In this Special Issue “New Trends in the Renin-Angiotensin-Aldosterone System in Cardiovascular Disease” the authors of eight experimental and clinical studies employed exquisite experimental tools with a common goal to comprehend causal links between disorders of RAAS and development of cardiovascular diseases, including those complicated by COVID-19 infection.

The study of Wesseling et al., “Mildly increased renin expression in the absence of kidney injury in the murine transverse aortic constriction model” concentrates on cardiovascular and biochemical effects of acute and chronic heart failure induced by transverse aortic constriction (TAC) in the mouse. The TAC model of kidney injury allows tracing dynamic changes in RAAS activity in the situation wherein the reduction of stroke volume precedes hypoperfusion of the kidney. Using 3D-echocardiography, and histological, biochemical and qPCR analyses, the authors show that TAC elicits significant increases in both renin concentration in the systemic blood and the number of renin positive cells in the renal glomeruli without apparent deterioration of kidney function. They conclude that the kidney dysfunction and increase of renin secretion during heart failure evoked by TAC cannot be exclusively explained by renal hypoperfusion, but involve more complex mechanisms.

The possibility that the central component of RAAS participates in augmented sodium intake in the renovascular hypertension caused by renal artery stenosis was explored by Lucera et al., “Revealing the central mechanisms involved in the increased sodium intake induced by water deprivation in renovascular hypertensive rats.” The authors demonstrated that separate blockade of central angiotensin AT1 or aldosterone receptors significantly reduces sodium appetite, and that combined blockade of both types of receptors abolishes enhanced sodium ingestion in this model. The authors emphasize the significant role of the brain RAAS in generating enhanced sodium intake in dehydrated rats.

The study of Huskova et al., “Increased endogenous activity of the renin-angiotensin system reduces infarct size in the rats with early angiotensin II-dependent hypertension which survive the acute ischemia/reperfusion injury” highlights the role of the RAAS in development of myocardial ischemia/reperfusion injury during Ang II-dependent hypertension. The authors report that Ang II-dependent hypertension induces cardiac hypertrophy and elevates Ang-(1–7) levels. They show that acute myocardial ischemia/reperfusion produces significant injury of the myocardium, elevates angiotensin levels and AT1 receptor expression, induces cardiac arrhythmia, and enhances mortality. Some of these effects are significantly reduced by administration of losartan, although blockade of AT1 receptors with losartan fails to reduce the ischemia-induced infarct size and plasma and heart levels of Ang II. The authors suggest that some beneficial effects of losartan in Ang II-dependent hypertension and in cardiac ischemia/reperfusion injury are mediated by extracardiac AT1 receptors.

The study of Baranowska et al., “Chymase dependent pathway of angiotensin II generation and rapeseed derived peptides for antihypertensive treatment of spontaneously hypertensive rats” evaluated the efficiency of chymase inhibitors (rapakinin and VWIS) in treatment of hypertension in spontaneously hypertensive rats (SHR). The authors report that rapkinin, which effectively interferes with Ang II generation independent from ACE inhibition, significantly reduces systolic blood pressure, elevates excretion of nitric oxide metabolites, and reduces the glomerulosclerosis index in SHR. The authors suggest that chymase inhibitors be considered as a valuable supplementary tool for treatment of hypertension, especially in so-called “ACE escape” phenomenon.

In the review article of Jędrusik et al., “The effect of antihypertensive medications on testing for primary aldosteronism,” the authors compared the impact of specific antihypertensive compounds in the evaluation of secondary hypertension caused by primary aldosteronism. Based on an extensive literature survey, the authors propose that calcium antagonists, alpha blockers, hydralazine and moxonidine exert less interference in on-drug evaluation for primary aldosteronism. The authors emphasize that testing other antihypertensive compounds should be analyzed with careful attention to biochemical parameters and after exclusion of confounding effects.

Hypertensive patients frequently do not adhere to prescribed therapies. Such nonadherence may cause severe complications. In their study Buffolo et al., “Assessment of anti-hypertensive drug adherence by serial aldosterone-to-renin ratio measurement,” evaluated usefulness of aldosterone-to-renin ratio (ARR) and ΔARR index to assess therapeutic compliance by hypertensive patients. Based on measurements of ARR and ΔARR before and after application of RAAS inhibitors in hypertensive and normotensive subjects, the authors postulate that ΔARR monitoring is an useful tool for therapeutic drug monitoring in patients suspected of non-adherence.

The significance of the local RAAS for the regulation of heart and lung functions has been extensively analyzed in the review article of Mascolo et al., “The role of renin-angiotensin-aldosterone system (RAAS) in the heart and lung: focus on COVID-19.” The authors draw attention to the role of angiotensin-converting enzyme 2 (ACE2) in the mechanism of COVID-19 entry into cells. They also discuss the potential usefulness of pharmaceutical compounds blocking effects of Ang II or promoting action of Ang-(1–7) in treatment of COVID-19 infections.

Similar problems were approached in the review by Ekholm and Kahan, “The impact of the renin-angiotensin-aldosterone system on inflammation, coagulation, and atherothrombotic complications, and to aggravated COVID-19 disease.” The authors provide deep analysis of the role of RAAS in pathophysiology of hypertension and consider usefulness of specific compounds interfering with the RAAS in various forms of hypertension, especially those associated with COVID-19 infection.

Collectively, the studies incorporated in the Special Issue “New Trends in the Renin-Angiotensin-Aldosterone System in Cardiovascular Disease” open new avenues for research and for improved programming of individualized therapies in cardiovascular diseases.

